# Synthesis, characterisation and thermo-physical properties of highly stable graphene oxide-based aqueous nanofluids for potential low-temperature direct absorption solar applications

**DOI:** 10.1038/s41598-021-94406-y

**Published:** 2021-08-16

**Authors:** Wisut Cham sa-ard, Derek Fawcett, Chun Che Fung, Peter Chapman, Supriya Rattan, Gerrard Eddy Jai Poinern

**Affiliations:** 1 Murdoch Applied Innovation Nanotechnology Research Group, Department of Physics, Energy Studies and Nanotechnology, Harry Butler Institute, Murdoch University, Murdoch, WA 6150 Australia; 2 School of Engineering and Energy, Murdoch University, Murdoch, WA 6150 Australia; 3 School of Molecular and Life Sciences, Faculty of Science and Engineering, Curtin University, Bentley, WA 6102 Australia

**Keywords:** Graphene, Graphene

## Abstract

Two types of highly stable 0.1% graphene oxide-based aqueous nanofluids were synthesised and investigated. The first nanofluid (GO) was prepared under the influence of ultrasonic irradiation without surfactant. The second nanofluid was treated with tetra ethyl ammonium hydroxide to reduce the graphene oxide to form reduced graphene oxide (RGO) during ultrasonic irradiation. The GO and RGO powders were characterised by various techniques such as field emission scanning electron microscopy, transmission electron microscopy, X-ray diffraction and Raman. Also UV–visible absorption spectroscopy was carried out and band gap energies were determined. Optical band gap energies for indirect transitions ranged from 3.4 to 4.4 eV and for direct transitions they ranged between 2.2 and 3.7 eV. Thermal conductivity measurements of the GO-based aqueous nanofluid revealed an enhancement of 9.5% at 40 °C compared to pure water, while the RGO-based aqueous nanofluid at 40 °C had a value 9.23% lower than pure water. Furthermore, the photothermal response of the RGO-based aqueous nanofluid had a temperature increase of 13.5 °C, (enhancement of 60.2%) compared to pure water, the GO-based aqueous nanofluid only displayed a temperature rise of 10.9 °C, (enhancement of 46.6%) after 20 min exposure to a solar irradiance of 1000 W m^−2^. Both nanofluid types displayed good long-term stability, with the GO-based aqueous nanofluid having a zeta potential of 30.3 mV and the RGO-based aqueous nanofluid having a value of 47.6 mV after 6 months. The good dispersion stability and photothermal performance makes both nanofluid types very promising working fluids for low-temperature direct absorption solar collectors.

## Introduction

Since the beginning of the twenty first century, there has been considerable scientific and industrial interest in multifunctional carbon-based nanometre scale materials like fullerenes, carbon nanotubes and recently graphene. This interest stems from the unique electronic, mechanical, optical and thermal properties exhibited by these nanometre scale carbonic materials^1,2^. Among these materials, graphene has attracted special interest due to its high mechanical strength, excellent thermal properties, outstanding carrier mobility, and unique chemical properties^3–6^. These unique properties arise from graphenes unique one-atom-thick planar array of tightly packed *sp*^2^ hybridised carbon atoms arranged in a two-dimensional (2D) honeycomb lattice structure^7^. Because of its unique properties, graphene was identified as an ideal candidate for several applications including electronic devices, sensors and photovoltaic systems^8–11^. And as a result, several physical and chemical processes were developed to produce large quantities of graphene at low cost. Physical processes include chemical vapour deposition (CVD), carbon nanotube unzipping, epitaxial growth, and micro-mechanical cleavage of highly ordered pyrolytic graphite^12–14^. While chemical processes include chemical synthesis from aromatic compounds, chemical reduction of graphene oxide, and direct exfoliation^15–17^. In recent years, newly emerging graphene oxide (GO), an oxidised derivative of graphene, has also attracted significant scientific and industrial interest due to its potential use in developing new sensors, electronic and optoelectronic devices, and organic solar cells^18–21^.

In spite of GO being a derivative of graphene, it still possesses similar properties like conductivity, mechanical strength and transparency to those of graphene^22–24^. GO consists of a two-dimensional graphene monolayer covalently bonded to a variety of surface oxygen-bearing functional groups, such as carbonyl, carboxyl, epoxide and hydroxyl. The basal plane of GO is decorated with epoxide and hydroxyl functional groups, while carbonyl and carboxylic functional groups are found attached to its edges^25^. The existence of these oxygen-bearing functional groups generates a two-dimensional array of *sp*^2^ and *sp*^3^ bonded atoms in the GO structure. The presence of these oxygen-bearing functional groups also promotes the interaction with a wide variety of organic and inorganic molecules to produce a range of GO-based materials with enhanced properties. The coverage of oxygen-bearing functional groups can vary depending on the degree of oxidation produced during manufacture. Extensive oxidation can reduce material properties, but the subsequent reduction of GO can restore property performance to values similar to graphene^26^. Typical methods for reducing graphene oxide include chemical reduction, thermal reduction and UV irradiation^27,28^.

Several interesting features of GO include fluorescence, large surface area, water-solubility and good colloidal stability. Its attractive amphipathic nature also permits easy dispersion in solvents like ethylene glycol (EG) and ionic liquids^29^. In addition to these properties, GO has superior mechanical properties, its surface can be easily modified, and it displays good biocompatibility^30^. GO can be generally manufactured by Hummers method, oxidative methods and structure refining techniques^31–33^. However, properties produced in the resulting GO’s are heavily dependent on the manufacturing process used. Thus, different manufacturing processes can produce GO’s with different optical and electronic properties resulting from the various oxygen-bearing functional groups generated during manufacture. For example, GO’s electronic structure depends on the stoichiometric carbon-to-oxygen atomic ratio resulting from the various oxygen-bearing functional groups present. Therefore, it is possible to transform insulating properties (wide band gap) to those of a semiconductor (finite band gap) or a graphene-like semi-metal by varying the fraction of *sp*^2^ and *sp*^3^ domains present in the GO structure^34^. Thus, prospective applications of graphene oxide are dependent on the material properties produced by the respective manufacturing process.

An interesting feature of graphene oxide is its atomically planar surface can act as a reaction site for the functionalization of oxygen-bearing groups and other active chemical species^35^. Moreover, GO’s optical properties and high thermal capacity have attracted the attention of researchers investigating methods of harvesting and converting abundant solar energy into electrical and thermal energy^36–38^. Among the various methods for exploiting solar energy, the most common method of harvesting and utilisation is via solar thermal collectors. Solar thermal collectors are designed to collect solar radiation, absorbed the radiation and converted into thermal energy, which is then transferred to a working fluid. Typically, solar thermal collectors consist of plates or tubes coated with a spectrally selective material that improves the absorption of solar irradiation^39^. Located within the plates or tubes is a circulating working fluid or heat transfer fluid that absorbs and transfers the thermal energy. The most attractive feature of solar thermal collectors is their energy output is totally renewable and does not produce greenhouse gas emissions. However, solar thermal collectors suffer from a number of shortcomings that include inefficient solar capture, low heat transfer rates to the working fluid and collector heat losses^40^. Thus, direct absorption solar collectors were developed to overcome these shortcomings by directly transferring solar energy into a circulating working fluid. The working fluid can flow in either an open-loop or closed-loop configuration. In the closed-loop configuration (higher energy applications), the working fluid flows through a heat exchanger circuit that separates it from a potable water circuit. Conventional working fluids used in heat exchanger circuits have included water, ethylene glycol, water/ethylene glycol mixtures and a range of oils. Unfortunately, these fluids displays low adsorptive properties over the solar spectrum (0.25 < λ < 2.5 µm) and makes them inefficient working fluids in direct absorption solar collectors^41^.

Globally, current research is aimed at developing new working fluids with enhanced optical and thermal properties since this will significantly improve the performance of direct solar absorption collectors. Several studies have shown the addition of low concentrations of micrometre scale particles (solid phase) to a conventional working fluid can significantly enhance its optical and thermal properties^42^. However, these two-phase fluids have a number of operational problems that include: (1) particle sedimentation resulting in reduced heat transfer rates; (2) high erosion rates caused by circulating particles; (3) particles tend to accumulate and block narrow flow channels; (4) increased flow resistance and larger pressure drops, and (5) improved thermo-physical properties of working fluid are achieved at larger particle concentrations, which in turn increases the above mentioned problems^43^. Because of these operational problems, two-phase working fluids containing micrometre scale particles have not gained acceptance. However, with the advent of multifunctional carbon-based nanometre scale materials, there has been a renewed interest in developing two-phase working fluids specifically designed for direct absorption solar collector applications. Importantly, when nanometre scale materials are dispersed in base fluids, they form colloidal suspensions known as nanofluids. Thus, avoiding many of the problems normally associated with two-phase fluids incorporating micrometre scale particles. Thus, in recent years several studies have regularly investigated the potential use of carbon-based nanometre scale materials like fullerenes, carbon nanotubes and graphene for inclusion in nanofluid formulations. In particular, GO has attracted significant interest due to the presence of hydrophilic groups on its surface and its excellent dispersion in water and alcohols, combined with attractive optical and thermal properties that make it a promising enhancement agent in water-based nanofluids^44–48^.

The present study, for the first time, has developed a facile approach for preparing well-dispersed GO and reduced graphene oxide (RGO) based aqueous nanofluids at room temperature. Tetra ethylene ammonium hydroxide (TEAH) was used as both reducing and stabilising agent. Commercially available GO powder and TEAH were the principal ingredients used in the straightforward ultrasound assisted procedure for producing aqueous- based nanofluids suitable for direct solar absorption collectors. The GO powders and GO-based aqueous nanofluids were characterised by several advanced characterisation techniques. Techniques like Fourier transform infrared spectroscopy (FT-IR), thermo-gravimetric analysis (TGA), X-ray diffraction (XRD), Raman spectroscopy and transmission electron microscopy (TEM) were used to analyse and compare the structure and composition of respective GO materials before and after treatment with TEAH. Ultraviolet visible (UV–visible) spectroscopy was used to monitor the degree of graphene oxide reduction. In addition, the UV–visible absorption spectra was used to generate Tauc plots with linear extrapolation to determine optical band gap energies of the GO and RGO powders. Furthermore, thermal conductivity, electrical conductivity and photo-thermal response of the respective nanofluids were also investigated.

## Results and discussions

In order to investigate the influence of tetra ethyl ammonium hydroxide (TEAH) during the transformation of graphene oxide (GO) to reduced graphene oxide (RGO) several electron microscopy and characterisation studies were undertaken. The following sections present the results of these investigations.

### Electron microscopy and nanofluid stability

**Figure 1:**
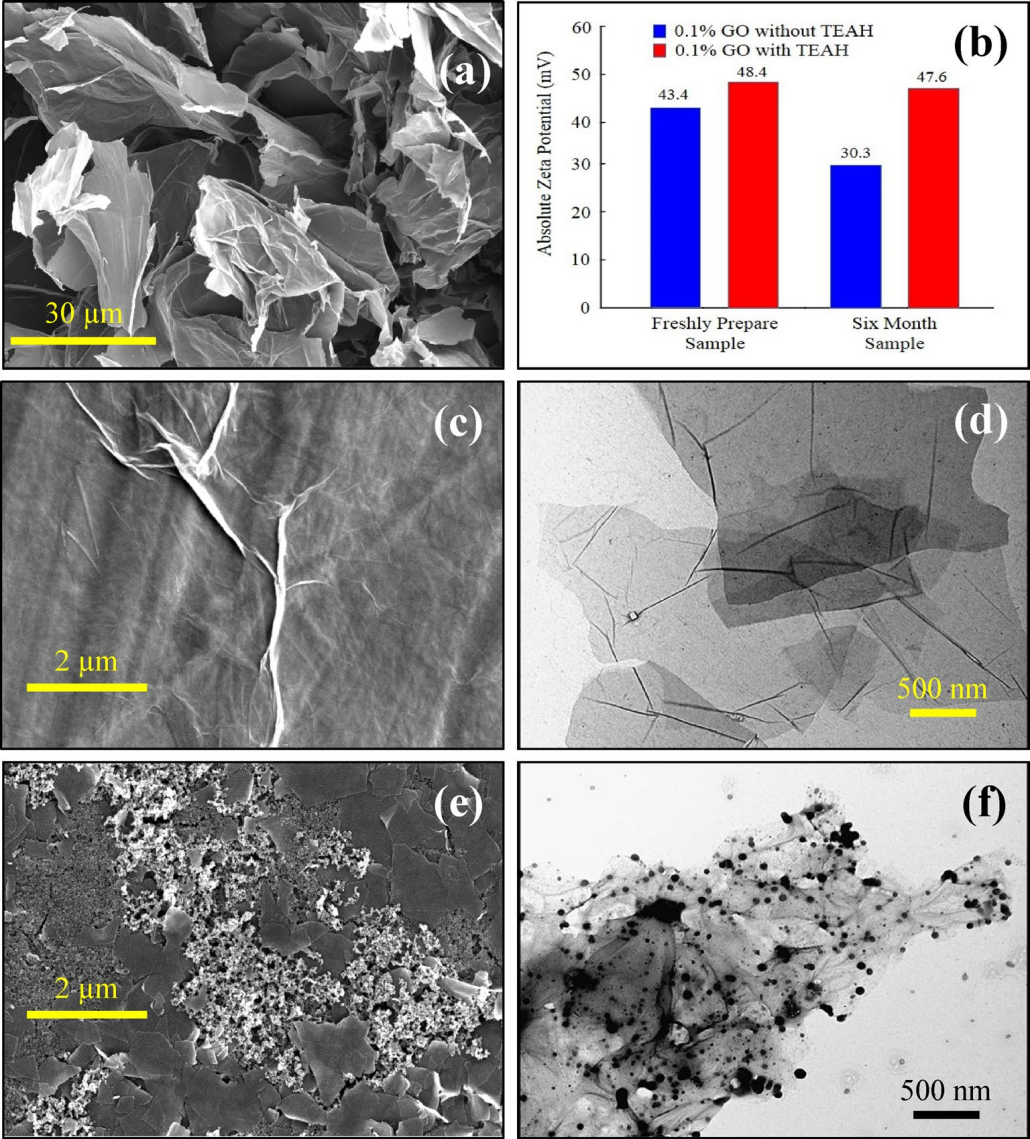
(**a**) FESEM image of GO prior to processing, (**b**) absolute zeta potential values for fresh nanofluid samples and those after 6 months storage, (**c**) FESEM and (**d**) TEM images of GO-based aqueous nanofluids and (**e**) FESEM and (**f**) TEM are images of RGO-based nanofluids.

Figure 1 presents representative electron microscopy images of dried nanofluid samples containing GO and RGO. Figure 1a presents a representative FESEM image of GO prior to processing. The image shows an agglomeration of large micrometre scale sheets displaying surface wrinkling and folding. Figure 1c presents a typical micrometre scale flake of GO, which displays some surface wrinkling. Figure 1d presents a representative TEM sample showing well dispersed and transparent sheets of GO. Stability measurements for these samples gave an absolute zeta potential of 43.4 mV (Fig. 1b). Typically, suspensions with absolute zeta potential values greater than 30 mV are physically stable, while suspensions below 20 mV have limited stability, and suspensions below 5 mV experience rapid aggregation. Thus, the GO-based aqueous nanofluid is physically stable and it was only after 6 months its zeta potential dropped down to 30.3 mV. In the case of the RGO-based aqueous nanofluid, the surface features of the flakes were different as seen in FESEM image presented in Fig. 1e and TEM image shown in Fig. 1f. The change in texture and surface features supports the results of the following characterisation studies that indicate oxygen-bearing functional groups and water molecules were expelled during chemical reduction by TEAH. Interestingly, the RGO-based aqueous nanofluid had a much larger initial absolute zeta potential value of 48.4 mV (Fig. 1b) and even after 6 months its value was still high with a value of 47.6 mV. This equated to a reduction of around 1.7% and clearly indicates the RGO-based aqueous nanofluid has very good long-term stability.

### FT-IR spectroscopy

**Figure 2:**
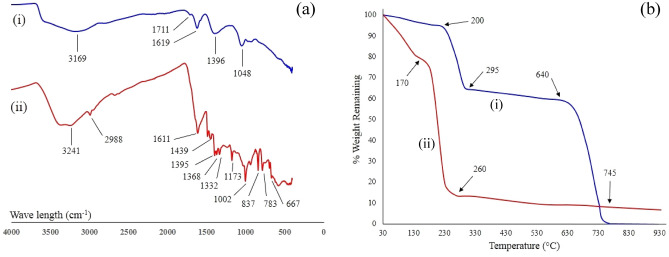
(**a**) FTIR spectra of (i) GO and (ii) RGO samples and (**b**) TGA of thermographs of (i) GO and (ii) RGO samples.

An FT-IR spectroscopy investigation was carried out on samples and Fig. 2a shows representative spectra of a GO and RGO based nanofluids. Figure 2a,i presents a representative spectrum of a GO-based aqueous nanofluid and shows a number of vibrational transitions. The spectrum displays a broad band at 3169 cm^−1^ that was identified as the O–H stretching vibrations of adsorbed water molecules and the presence of structural OH groups^49^. In the middle of the spectra are two other bands, the weak 1711 cm^−1^ and much stronger 1619 cm^−1^. The weak 1711 cm^−1^ band was assigned to C=O stretching vibrations of carbonyl groups, while the much stronger band located at 1619 cm^−1^ was assigned to C=O stretching vibrations of carboxylic and/or carbonyl functional groups. However, some studies have suggested the 1619 cm^−1^ band could be the result of water bending modes^50^ and others believe it to be the aromatic C=C bond^51^. The band identified at 1396 cm^−1^ could be the result of C–OH bending vibrations or O–H deformation, while the band at 1048 cm^−1^ was assigned to C–O stretching vibrations. The use of TEAH to produce RGO-based aqueous nanofluids produced noticeable differences in the spectra as seen in Fig. 2a,ii. The band located at 3241 cm^−1^ was identified as O–H stretching vibrations of adsorbed water molecules and also indicated the presence of structural OH groups.

Moving to the right, the next band encountered is located at 2988 cm^−1^ and was assigned as C–H stretching vibration. Next are the bands located at 1611 cm^−1^ and 1439 cm^−1^, which were assigned as C=C stretching vibration and C–H bending vibrations respectively. Also present in the spectra, which was not seen in the GO samples were the bands located at 1395, 1368 and 1332 cm^−1^ and were assigned as –NO_2_ (aliphatic). The next band located at 1173 cm^−1^ was assigned as C–N stretching vibration and neighbouring 1002 cm^−1^ band was assigned to C–O stretching vibrations. The remaining bands located at 837, 783 and 667 cm^−1^ were assigned as C–H bending.

### Thermo-gravimetric analyses (TGAs) of GO and RGO powders

The thermal stability and decomposition of GO and RGO powder samples was determined using TGA. A representative thermograph for a GO sample is presented in Fig. 2b–i and shows a three-step weight loss pattern that indicates a complicated decomposition process occurring over the 30–800 °C temperature range. The initial decomposition takes place between 30 and 200 °C. During this stage, there was a weight loss of around 6% and resulted from the evaporation of absorbed water molecules. Then starting at around 200 °C and ending at 295 °C there was a rapid weight loss. This weight loss was estimated to be around 29% and resulted from the pyrolysis of labile oxygen-bearing functional groups and oxygen-containing groups like CO, CO_2_ and H_2_O. From 295 °C up to around 640 °C there was an additional weight loss of around 8%, indicating the further removal of smaller amounts of functional groups. Then around 640 °C there is a rapid weight loss of approximately 56%, and by 745 °C there was virtually no weight remaining. A representative thermograph for a RGO sample is presented in Fig. 2b-ii and shows a two-step weight loss pattern that is quite different from the GO sample. The first step starts at 30 °C and continues to about 130 °C and is accompanied by a weight loss of around 19%. This weight loss was credited to the evaporation of absorbed water molecules. The second step occurred around 200 °C and resulted in a weight loss of approximately 71%. This dramatic step was due to the pyrolysis of the labile oxygen-containing groups like CO, CO_2_ and H_2_O, and the decomposition of absorbed TEA + species present on the RGO sample. FTIR spectra presented in Fig. 2a-ii for RGO revealed the presence of oxygen-containing functional groups. These functional groups provide a large number of potential reactive sites that increase the ion exchange capacity between TEA^+^ ions in solution during treatment and H^+^ ions present in the functional groups still present on the surface of the RGO. Similar studies by Chang et al*.* using GO to remove tetramethylammonium hydroxide (TMAH) from water found the oxygen-containing groups present on the surface of GO had a strong affinity for absorbing TMAH. Their study also revealed the ion exchange between TMA^+^ ions in solution and H^+^ ions of the oxygen-containing functional groups present on the surface of GO, as well as electrostatic attraction, were the factors contributing to the adsorption mechanism^52^.

### X-ray diffraction spectroscopy

**Figure 3:**
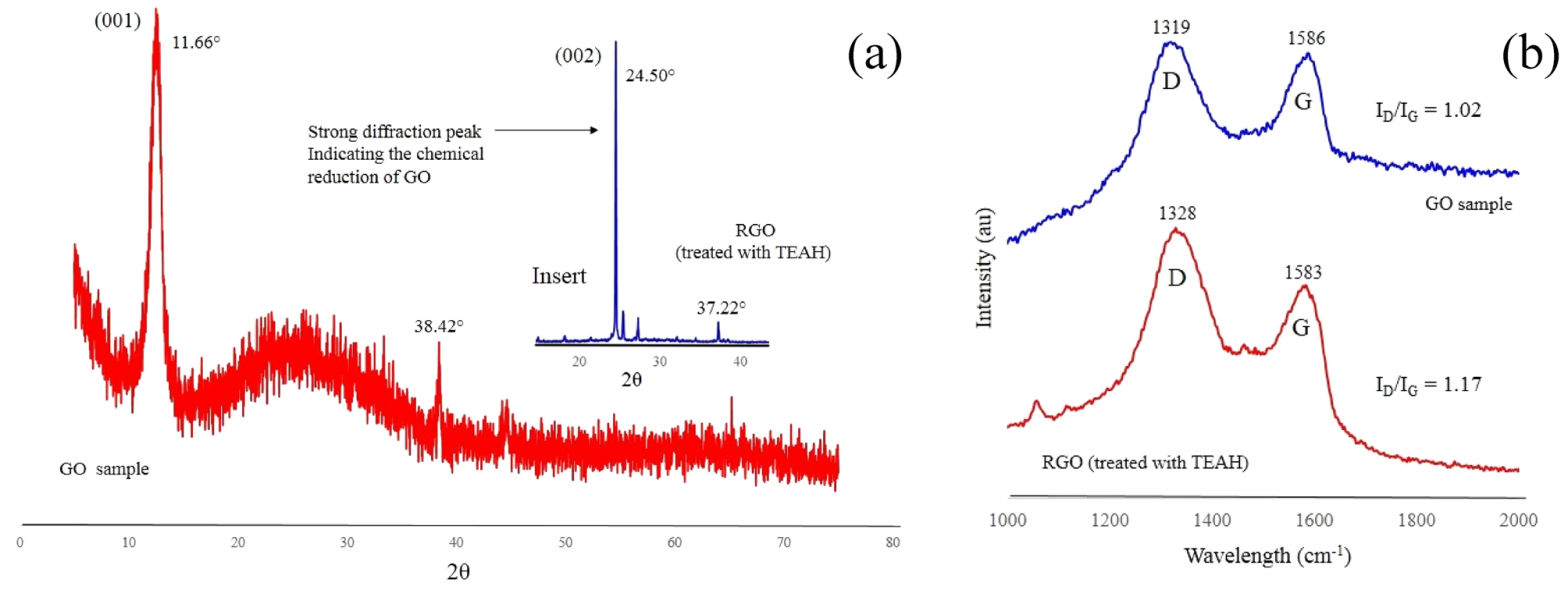
(**a**) XRD patterns and (**b**) Raman spectra of GO and RGO samples.

XRD was used to investigate the crystalline structure present in the respective samples. Representative GO and RGO powder samples are presented in Fig. 3. A typical GO XRD pattern is presented in Fig. 3a and displays the characteristic diffraction peak located at 2θ = 11.66°, which was attributed to the (001) crystalline plane of GO. The corresponding inter-layer spacing (d spacing) of powder samples were calculated using Eq. (1) below:

1 $${d}_{space}=\frac{\lambda }{ 2\mathrm{ sin}\theta }$$

where d_*space*_ is the inter-layer spacing, *λ* is the X-ray source wavelength, and θ is half of the corresponding peak diffraction angle. The calculated inter-layer spacing for the (001) peak identified in the GO sample was found to be 0.758 nm. The diffraction pattern for the RGO sample revealed the (001) peak had disappeared and another, more intense diffraction peak appeared at a 2θ value of 24.50°. The intense peak was identified as the (002) peak for graphite, and the subsequent inter-layer spacing was calculated to be 0.363 nm, which is similar to the inter-layer spacing for pure graphite (0.334 nm). The larger inter-layer spacing of 0.758 nm for the GO sample results from the inclusion of oxygen-bearing functional groups and water molecules between the graphene layers produced during oxidation. The variation in the amount of oxidation taking place also explains the difference in 2θ (10°–12.5°) reported in the literature^53,54^. Importantly, the presence of the (002) peak and the inter-layer spacing of 0.363 nm in the RGO sample confirms TEAH does reduce GO. Thus, giving it a more graphitic-like character. The perpendicular dimension or average crystallite size (D_(*hkl*)_) that contains the graphitic ordering is expressed by the Debye–Scherer equation:

2 $${D}_{(hkl)}=\frac{k\lambda }{\upbeta {\mathrm{cos}\theta }_{(hkl)}}$$

where *λ* is the wavelength of the monochromatic X-ray beam and crystallite shape constant *k*, which is 0.89 for spherical crystals with cubic unit cells. And *β* is the Full Width at Half Maximum (FWHM) of the peak at the maximum intensity, θ_(*hkl*)_ is the peak diffraction angle that satisfies Bragg’s law for the (*hkl*) plane and D_*(hkl)*_ is the crystallite size. An estimate of the mean GO crystallite size for the (001) peak was calculated to be 12.53 nm. While the mean crystallite size for the RGO sample using the (002) peak was estimated to be 1.16 nm. The average number of graphene layers (n) per domain was calculated using results produced from Eqs. (1) and (2) using the expression (3) below:

3 $$n=\left(\frac{{D}_{(hkl)}}{{d}_{space}}+1\right)$$

The results of Eq. (3) reveal a significant decrease in the number of graphene layers (*n*) present in the crystallite after chemical reduction with TEAH. The GO sample produced an *n* value of 18, while the RGO sample had a much lower n value of 4. The reduction in the n value can be attributed to the elimination of oxygen-bearing functional groups and water molecules between the graphene layers during chemical reduction by TEAH. Also, the in-plane crystallite size (*L*_*p*_) was expressed in terms of the in-plane periodicity peak in the respective XRD patterns using Eq. (4) below:

4 $${L}_{p}=\frac{1.84\lambda }{\beta \mathrm{cos}\theta }$$

where θ is the diffraction angle of in-plane periodicity peak. From XRD pattern, the GO sample’s in-plane periodicity peak was located at 2θ = 38.42° and the in-plane crystallite size was calculated to be 38.22 nm. The RGO sample had an in-plane periodicity peak locate at 2θ = 37.22° that was used to calculated an in-plane crystallite size of 21.69 nm. The results of the XRD analysis are tabulated in Table 1 and reveal a decreasing trend in inter-layer spacing (d_*space*_), crystallite sizes (D_(*hkl*)_) and in-plane crystallite sizes (*L*_*p*_) for the RGO sample. This decreasing trend results from the removal of oxygen-bearing functional groups and water molecules that occur during the TEAH reduction of GO.

**Table 1:** Experimental parameters derived from XRD and Raman spectroscopy analysis.

Technique	XRD					Raman		
Parameters sample	2θ (°)	d_*space*_ (nm)	D (nm)	Layers (n)	*L*_*p*_ (nm)	D band (cm^−1^)	G Band (cm^−1^)	I_D_/I_G_
GO	11.66	0.758	12.53	18	38.22	1319	1586	1.02
RGO	24.50	0.363	1.16	4	21.69	1328	1583	1.17

### Raman spectroscopy

Raman spectroscopy is a widely used technique to investigate and structurally characterise graphene-based materials. Figure 3b presents representative Raman spectra of GO and RGO samples. The spectra for a GO sample is characterised by two main prominent peaks, a G band (1586 cm^−1^) and disorder-induced D band (1319 cm^−1^). The G band is always between 1500 and 1630 cm^−1^ for all poly-aromatic hydrocarbons and results from the in-plane vibration of *sp*^2^ carbon atoms (E2g symmetry mode)^55^. While the D band (the symmetric A1g mode) results from the disordered structures produced by extensive oxidation present in GO samples^56^. The RGO sample spectra also exhibits both G (1583 cm^−1^) and D (1328 cm^−1^) bands. Inspection of Fig. 3b and Table 1 reveals the ratio intensities (I_D_/I_G_) changes from 1.02 in the GO sample to 1.17 in the RGO sample. Since the intensity ratio (I_D_/I_G_) is inversely proportional to the average size of the *sp*^2^ domains, the higher (I_D_/I_G_) ratio for the RGO sample indicates a smaller number of *sp*^2^ domains and a larger number of new graphitic domains being created^56^. This result confirms the results of Stankovich et al., that GO was indeed reduced by the presence of TEAH during synthesis and supports the XRD results, which indicated oxygen-bearing functional groups and water molecules were expelled during the chemical reduction of the GO structure.

### UV–visible absorption spectra and estimated optical band gap energies

**Figure 4:**
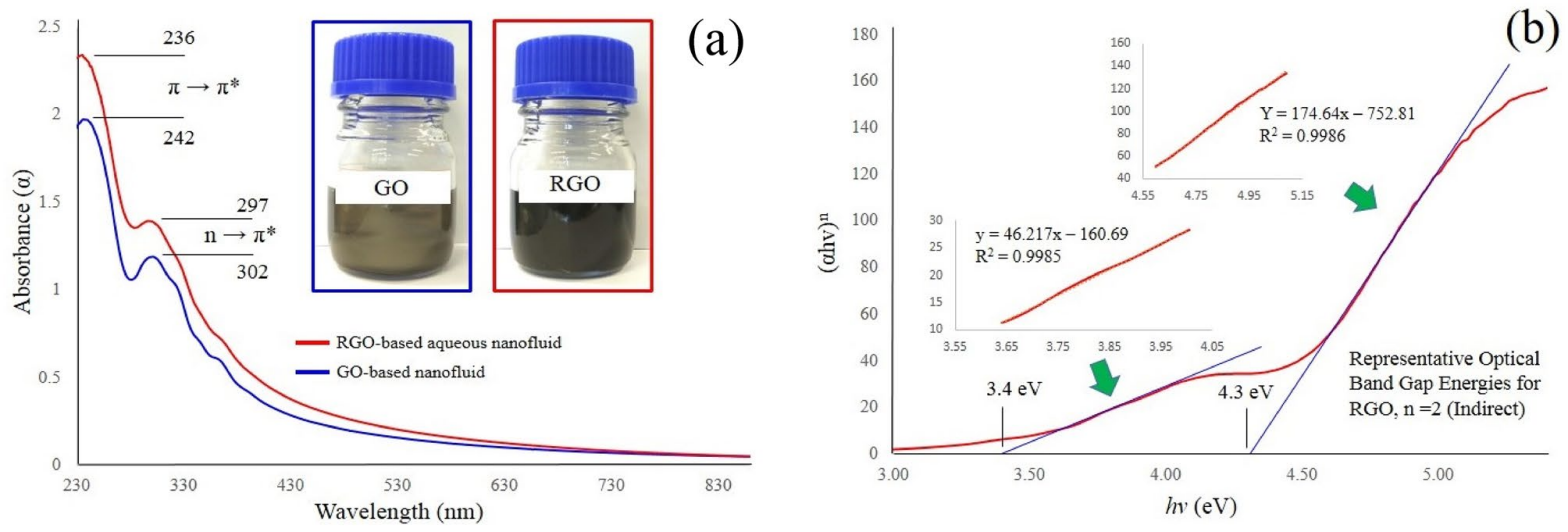
(**a**) UV–Vis absorption spectrum of GO and RGO based aqueous nanofluids and (**b**) Tauc plot of (*αhv*)^n^ versus photon energy (*hv*) for a RGO based aqueous nanofluid.

The UV–Visible absorption spectrum recorded for GO and RGO based aqueous nanofluids is shown in Fig. 4a. The absorption peaks are typical of single-layer GO suspensions reported in the literature. The spectra is characterised by a main absorption peak, and a smaller shoulder peak. The main absorption peak for the nanofluids are located at wavelengths of 242 nm (GO) and 236 nm (RGO), and correspond to the π → π* transition of *sp*^2^ poly-aromatic (C–C bonds) carbon structures^57^. The absorption peaks of the GO–based aqueous nanofluids are also blue shift when compared to graphene (270 nm). The shoulder absorption peak for the nanofluids are located at wavelengths of 302 nm (GO) and 297 nm (RGO), and correspond to the n → π* electron transitions in carbonyl and carboxyl (C=O bonds) functional groups^58,59^. This result was also confirmed by FTIR spectroscopy analysis which revealed the presence of oxygen functional groups. The absorption spectra also revealed a small shift in peak positions (5–6 nm), with wavelengths of the peaks being slightly less for RGO nanofluids. Thus, the changes in the absorption spectra indicate the use of TEAH to produce RGO has modified the chemical structure of absorbing species present on the original GO sheets. Interestingly, XRD data also confirmed the reduction of oxygen-bearing functional groups and water molecules between the graphene sheets when TEAH was usd to reduce GO.

The π → π* and n → π* transitions were clearly defined for GO and RGO based aqueous nanofluids, and accordingly the optical band gap energies were calculated from the respective UV–visible absorption spectra and Tauc plots with linear extrapolation. The optical band gap energy (E_g_) was extracted from the absorption coefficient using Tauc’s equation diffraction (XRD)Spectrosc^60^:

5 $${\left(\alpha hv\right)}^{n}=B\left(hv-{E}_{g}\right)$$

where *α* is the absorption coefficient, *hv* is the incident photon energy, and *B* is material dependent constant, while parameter n denotes the character of the optical transition. For example, n = 2 represents indirect (I) transitions and n = ½ represents direct (D) transitions. Thus, plotting (*αhv*)^n^ against the incident photon energy and extrapolating the linear region of the curve to the x-axis will give a value for the optical band gap energy. However, understanding the electronic band structure of GO is difficult due to its amorphous nature and non-uniform oxidation levels that prevent a sharp adsorption edge in the Tauc plot. Instead, we see an approximate optical band gap energy range as seen in Fig. 4b and Table 2.

**Table 2:** Band gap energy values (*Eg*), indirect (I) and direct (D), obtained from Tauc plots of GO and RGO based aqueous nanofluids.

Sample	Optical band gap energies (eV)	
	n = 2 (I)	n = ½ (D)
GO	3.4–4.4	2.2–3.4
RGO	3.4–4.3	2.5–3.7

Similar variations in the optical band gap energy have also been reported by Hsu et al*.* (2.9–4.4 eV)^61^ and Kumar et al*.* (2.9–3.7 eV)^62^. The variation in optical band gap energies reported in the literature results from different processing factors such as time and the degree of oxidation, chemical functionalization, which can significantly influence structural, electronic and optical properties of GO^63^.

### Thermal conductivity and viscosity variations with increasing nanofluid temperature

**Figure 5:**
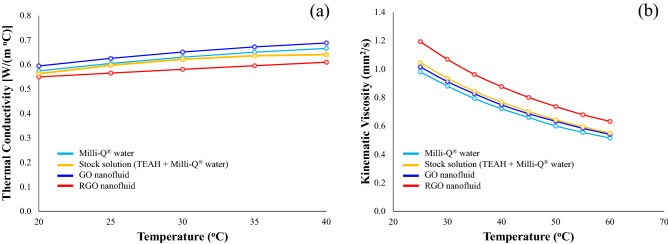
(**a**) Variation in thermal conductivity of GO and RGO nanofluids with temperature and (**b**) variation in nanofluid viscosity with temperature.

The thermal conductivity of Milli-Q® water as a function of temperature was measured, and the results were compared with literature values in order to establish and confirm the reliability and accuracy of the measurements^64^. The experimental results show good agreement with the reference data, since all measurements are within the maximum relative standard deviation of 3.5%. This result also verifies the accuracy of the KD2 Pro Thermal Properties Analyser and the reliability and consistency of the experimental procedure used for measuring the thermal conductivity of water-based fluids and nanofluids. As expected, the thermal conductivity of Milli-Q® water increased with increasing temperature (20–40 °C) as seen in Fig. 5a. Before nanofluid measurements were made it was necessary to determine the influence of TEAH on the thermal conductivity of Milli-Q® water. Therefore measurements of the stock solution (TEAH + Milli-Q® water) were carried out and discovered the supressing influence of TEAH on the thermal conductivity of the stock solution. Overall, the stock solution had a lower thermal conductivity than the Milli-Q® water. Initially, the reduction in thermal conductivity was 2.2% at 20 °C and steadily decreased to 4.3% at 40 °C as seen in Fig. 5a. However, the GO-based aqueous nanofluid displayed a definite improvement in thermal conductivity. At 20 °C the nanofluids thermal conductivity was 0.594 W m^−1^ °C, which equated to an improvement of 5.33% compared to pure Milli-Q® water. With increasing temperature, the thermal conductivity increased to 0.668 W m^−1^ °C. This equated to an enhancement in thermal conductivity of 9.51% at 40 °C. Conversely, the thermal conductivity of the RGO-based aqueous nanofluid was overall lower than the Milli-Q® water. At 20 °C the nanofluid was 2.36% lower and at 40 °C it was 9.23% lower compared to pure Milli-Q® water. This was due to the presence of TEAH suppressing the thermal conductivity of the RGO-based aqueous nanofluid, as was seen in Fig. 5a. Similar suppression behaviours have also been reported for other dispersants and surfactants used to stabilise nanofluids^65^.

Several researchers have also reported improving nanofluid thermal conductivities with increasing temperature, and suggest increasing Brownian motions of the suspended nanoparticles as a possible mechanism^66,67^. Since increasing Brownian motions tends to promote micro-convection, which in turns enhances local mixing^68,69^. In the case of the GO-based aqueous nanofluid there is clearly an increase in thermal conductivity with temperature and this mechanism would explain why it is occurring. However, in the case of the RGO-based aqueous nanofluid there was no improvement in thermal conductivity and instead there was a consistent suppression across the entire temperature range when compared to Milli-Q® water. This suggests the presence of TEAH in solution coates the suspended nanoparticles and suppresses thermal interactions between the nanoparticles and the surrounding fluid environment to significantly reduce the thermal conductivity of the nanofluid.

Figure 5b presents the results of measuring kinematic viscosity of Milli-Q® water, stock solution, and GO and RGO based aqueous nanofluids as a function of temperature. Inspecting Fig. 5b reveals the viscosity of all fluids decreases with increasing temperature. The presence of TEAH in the water-based stock solution results in a 6.7% increase in viscosity compared to Milli-Q® water over the temperature range. While the kinematic viscosity of the GO-based aqueous nanofluid at 20 °C is 1.015 mm^2^/s, which is 3.6% higher than Milli-Q® water and at 60 °C is slightly higher at 5.1%. The difference in viscosity between the two fluids results from graphene oxide flakes being present in the nanofluid. The RGO-based aqueous nanofluid at 20 °C had a kinematic viscosity of 1.193 mm^2^/s, which was 21.7% higher than Milli-Q® water and at 60 °C is slightly higher at 22.7%. The higher viscosity seen in the RGO-based aqueous nanofluid resulted from two factors. The first factor being the presence of TEAH in the base fluid. And the second factor was the increase in surface area of the well-dispersed and suspended nanoparticles. Other studies have also confirmed that well-dispersed carbon-based nanofluids display higher viscosities compared to nanofluids with agglomerated and clustered nanoparticles^70^. This was also reflected in the nanofluid stability measurements, which revealed the RGO-based aqueous nanofluid had an absolute zeta potential value of 47.6 mV even after 6 months in storage. Unlike the GO-based aqueous nanofluid which saw a reduction in the zeta potential of around 30% over the same 6 month period.

### Photothermal response of GO and RGO based aqueous nanofluids

**Figure 6:**
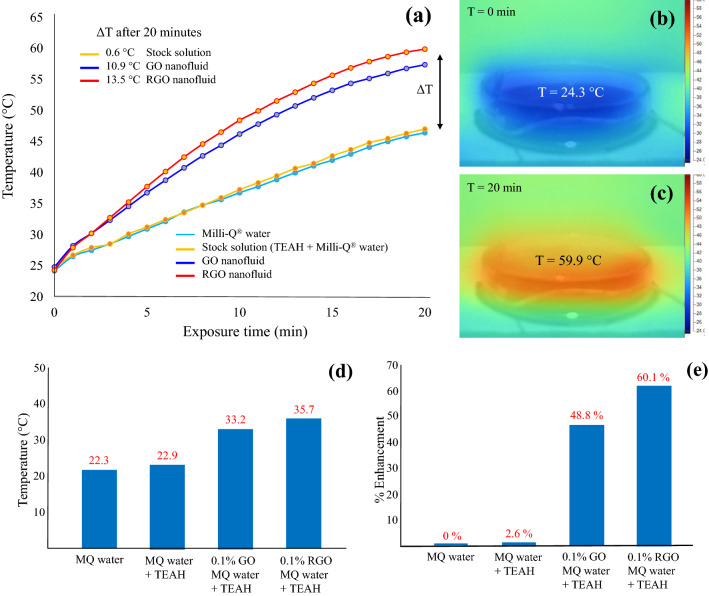
(**a**) Photothermal response of base fluids, GO and RGO nanofluids and (**b**,**c**) thermographs of RGO-based nanofluid taken over 20 min while exposed to solar irradiation. The temperature rise profile of the various nanofluids are shown (**d**) and (**e**) represents the % of phtothermal response enhancement with respct to MQ water.

The photothermal response of Milli-Q® water, stock solution, and GO and RGO based aqueous nanofluids as a function of time when exposed to a solar irradiance of 1000 W m^−2^ is presented in Fig. 6a. Inspection of Fig. 6a reveals the presence of TEAH in the stock solution gave a small photothermal response when compared to the pure Milli-Q® water control solution. Even after 20 min of exposure to solar irradiation the stock solution was only 0.6 °C higher than the Milli-Q® water control solution. Therefore, the significant photothermal response seen in the GO and RGO based aqueous nanofluids was the result of their respective solid components. For instance, after 20 min exposure to solar irradiation the GO-based aqueous nanofluid reached a temperature of 57.4 °C and was 10.9 °C higher than the pure Milli-Q® water control solution. While the maximum temperature reached by the RGO-based aqueous nanofluid reached 59.9 °C as seen in Fig. 6b,c and equated to a temperature enhancement of 13.5 °C. The impact of TEAH and GO/RGO on addition to MQ to create nanofluids, when examined in light of enhancement (ΔT °C) is further shown in Fig. 6d,e. The 20 min exposure was translated into a raw percentage rise of 46.6% (GO without TEAH) and 60.2% (GO with TEAH). These results clearly indicate the presence of small quantities of GO or RGO can significantly improve the photothermal response of the base fluid. Similar studies by other researchers have also shown that small quantities of various forms of carbon-based materials can also promote temperature enhancements comparable to the results of this study. For example, Han et al., using a 7.7% (vol.) carbon black-based aqueous nanofluid was capable of producing a 7.2 °C temperature enhancement compared to pure water^71^. While Poinern et al., using a 0.04% (vol.) carbon nano-sphere based nanofluid reported an 8.1 °C temperature enhancement compared to pure water^72^. Both GO and RGO based aqueous nanofluids synthesised in the present work have shown good photothermal responses. In particular the RGO-based aqueous nanofluid exhibits a higher temperature enhancement (13.5 °C) making it an ideal candidate for solar thermal applications. Furthermore, TEAH not only reduces GO to form a more graphitic material (RGO), TEAH also assists in producing a highly stable nanofluid. Even after 6 months its zeta potential was 47.6 mV and showed no signs of sedimentation.

## Conclusion

GO was reduced by TEAH under the influence of ultrasonic irradiation to produce RGO. Both GO and RGO were characterised using a variety of advanced characterisation techniques to determine the initial physiochemical properties of GO and to determine resulting graphitic nature of the synthesised RGO. Characterisation studies revealed oxygen-bearing functional groups and water molecules were expelled during the chemical reduction of the initial GO structure. Changes identified in the FTIR and XRD studies, and small changes in the optical band gap also confirmed structural modifications and differences in functional groups were generated by TEAH as it reduced GO to form RGO. Thermal conductivity measurements revealed GO based aqueous nanofluids had a 9.5% enhancement at 40 °C compared to Milli-Q® water. Whereas RGO based aqueous nanofluids showed a decrease in thermal conductivity of around 9.2% compared to Milli-Q® water. However, the RGO-based aqueous nanofluid showed the largest photothermal enhancement of 13.5 °C, (photo-enhancement of 60.2%) while GO-based aqueous nanofluid only displayed a 10.9 °C rise, equivalent to a photo-enhancement of 46.6% compared Milli-Q® water after 20 min exposure to a solar irradiance. Both nanofluid types were found to have good long-term stability, with the GO-based nanofluids having a zeta potential of 30.3 mV and RGO-based nanofluids having a value of 47.6 mV after 6 months. Therefore, the good dispersion stability and photothermal performance makes both nanofluid types very promising working fluids for low-temperature direct absorption solar collectors. However, the higher thermal conductivity (9.5%) of the GO-based aqueous nanofluid makes it the most promising candidate for direct absorption solar collectors.

## Methods

### Materials

Graphene oxide (GO) powder (product number: GNOS0010) was purchased from ACS Material, LLC (Pasadena, California, United States of America) and Tetra ethyl ammonium hydroxide [TEAH, C_8_H_21_NO, (35% in water)] was purchased from Tokyo Chemical Industry Co., Ltd. (Kita-Ku, Tokyo, Japan). All chemicals and solvents were analytical grade and used without further purification. All aqueous-based solutions used in the present study were produced from Milli-Q® water (10 MΩ cm^−1^) generated from a Milli-Q® Reagent water system supplied by the Millipore Corporation.

### Synthesis of GO-based aqueous nanofluids

Two types of GO-based nanofluids were produced. The first type was a GO-based aqueous nanofluid. While the second type was also a GO water-based nanofluid, but this time utilising TEAH to first reduce the GO to form RGO and then promote greater nanofluid stability.

#### Synthesis of 0.1% GO-based aqueous nanofluid (without TEAH)

The procedure begins by first adding 0.1 g of GO powder into a mortar and then adding a 2 ml solution of Milli-Q® water. The mixture is then ground for five minutes to produce a smooth paste. The paste was then added to a 100 ml solution of Milli-Q® water. The solution was then sonicated for 10 min using an ultrasonic processor (Hielscher UP400S) set at a power level of 400 W. After ultrasonic treatment, the resulting greyish tinted brown solution (as seen in Fig. 4a insert) was stored at room temperature ready for further studies.

#### Synthesis of 0.1% RGO-based aqueous nanofluid (with TEAH)

Initially, in a 100 ml Schott bottle, TEAH (20% by volume) was added and mixed with Milli-Q® water to make a 100 ml stock solution. Then 0.1 g of GO powder was placed into a mortar and then 2 ml of stock solution was added. The mixture is then ground for five minutes to produce a smooth paste. The paste was added to a 100 ml beaker containing a solution of 10 ml of stock solution and 90 ml of Milli-Q® water. The combined solution was then sonicated for 10 min using the ultrasonic processor set at a power level of 400 W. After ultrasonic treatment, the resulting black solution (as seen in Fig. 4a insert) was stored at room temperature ready for further studies.

### Characterisation of powder samples and fluids

#### Electron microscopy studies

Two electron microscopy studies were undertaken. The first study was a bright field transmission electron microscopy (TEM) investigation of the size, shape and topography of the various GO and RGO powder samples. The study was carried out using a Tecnai G2, FEI, (Electron Optics, USA) microscope operating at 100 kV. The second study used a high resolution field emission scanning electron microscope (FESEM: FEI-Verios 460) operating at 5 kV, with 0.10 nA current and operating under secondary electron mode. The FESEM was also used to examine the physical structure and topography of the GO and RGO powder samples. Prior to analysis, dried powder samples were deposited on carbon tape covered SEM holders and then sputter coated (E5000, Polaron Equipment Ltd.) with a 2 nm layer of platinum to prevent charge build up.

#### Fourier transform infrared spectroscopy (FT-IR)

FT-IR studies were undertaken to identify functional groups and their respective vibration modes present in the samples using a PerkinElmer FT-IR/NIR Spectrometer Frontier with Universal signal bounce Diamond ATR attachment. FT-IR spectra were recorded in the scanning range from 400 to 4000 cm^−1^ with a resolution step of 1 cm^−1^.

#### Thermo-gravimetric analysis (TGA)

TGA was carried out to evaluate the thermal stability of the GO and RGO powder samples using a Perkin Elmer simultaneous thermal analyser STA 8000. Thermal stability was carried out in air at a flow rate of 20 ml min^−1^, over a temperature range of 30–1000 °C with an incremental heating rate of 25 °C min^−1^.

#### X-ray diffraction (XRD) spectroscopy

The crystalline structure of the GO and RGO powder samples were characterised using X-ray diffraction spectroscopy. X-ray powder diffraction patterns were recorded using a GBC® eMMA X-Ray Powder Diffractometer (Cu Kα = 1.54056 Å radiation source) operating at 35 kV and 28 mA. Diffraction patterns were collected over a 2θ range starting at 5° and finishing at 75° with an incremental step size of 0.02° and an acquisition speed of 2° min^−1^. Sample preparation consisted of taking two to three drops from each glass vial containing a particular liquid samples. The drops were deposited and spread over individual glass microscope slides, which were then dried under vacuum for a period of 24 h at a temperature of 40 °C. The observed Bragg peak positions in the patterns were compared with those reported in the ICDD (International Centre for Diffraction Data) databases and the appropriate Miller indices were assigned to the respective peaks.

#### Raman spectroscopy

Raman spectroscopy measurements were carried out by a LabRAM 1B Raman spectrometer using a 632.82 nm Helium Neon laser light source with a spectral resolution of 1 cm^−1^. This technique was used to determine molecular vibrations and chemical compositions of the various samples.

#### UV–visible spectroscopy

UV–visible spectroscopy was used to assess the chromophores present in the GO and RGO samples and determine the optical band gap energies of the respective samples. Samples were scanned using an Agilent/HP 8453 UV–visible Spectrophotometer over a spectral range between 190 and 1100 nm, with a range resolution of 1 nm.

#### Photo-thermal response of GO and RGO based aqueous nanofluids

The photo-thermal response of the various fluids and nanofluids was carried out in a specifically designed solar simulator. The solar simulator light source was produced by a Philips 13096 ELH (120 V, 300 W-G5D) light tube. The simulator was adjusted and calibrated using a LI-200 R Pyranometer to produce a solar irradiance of 985 W m^−2^. A covered Petri dish, containing 25 g-of the sample to be tested was placed over insulation material at a distance of 25 cm from the illumination source. The temperature of the samples were monitored using a Digitech QM-1600 m with the thermocouple inserted into the fluid, while thermal images were recorded using a hand held thermal camera (Fluke Ti 25). Three sets of temperature measurements were carried out and recorded in real time, with the mean value being used. Measurements were carried out at room temperature, which was typically around 25 °C.

#### Thermal conductivity of nanofluids

Thermal conductivity measurements were carried out using a KD2 Pro Thermal Properties Analyser (Decagon Devices, Inc.). The instrument is based on the transient hot wire method and uses a single-needle sensor probe for heating and monitoring temperature. The thermal conductivity range of the instrument is between 0.02 and 2 W m^−1^ K^−1^. The probe (1.3 mm in diameter and 60 mm long) was vertically immersed in the centre of the sample. For each sample, measurements were taken at every 15 min, and the thermal conductivity was determined from the mean of ten readings at the same temperature. The sample container was immersed in a temperature controlled water circulating bath [GD 120 Grant Instruments (Cambridge) Ltd.] to ensure the constant temperature measurements. Initially, the probe was calibrated against pure water within the temperature range of 20–40 °C and produced a maximum relative standard deviation of 3.5%.

#### Nanofluid stability measurements

In order to quantitatively determine long-term stability of the nanofluids their zeta potentials (ζ) were measured using a Zetasizer Nano-ZS (Malvern Instruments, England). The samples were measured at 25 °C and measurement accuracy was within 2%.

#### Nanofluid viscosity measurements

The kinematic viscosity of the respective nanofluids was measured using a Ubbelohde Viscometer (SCHOTT-GERATE GmbH, Germany). The capillary based method was used to measure viscosity in a temperature range between 25 and 60 °C with incremental temperature reading taken every 5 °C. Temperature regulation of the viscometer was maintained using a temperature-controlled glass water bath [GD 120 Grant Instrument (Cambridge) Ltd.]. The mean of five measurements at each temperature was used to ensure reliable and accuracy of each measurement.
